# Supplementary material for: Ethyl Caffeate Can Inhibit Aryl Hydrocarbon Receptor (AhR) Signaling and AhR-Mediated Potentiation of Mast Cell Activation
Source: Int J Mol Sci. 2023 Jun 10;24(12):9997. doi: 10.3390/ijms24129997 (PMC10297944; doi:10.3390/ijms24129997)
Supplement: Supplementary file 1 [file ijms-24-09997-s001.zip › ijms-2427264-supplementary.pdf]

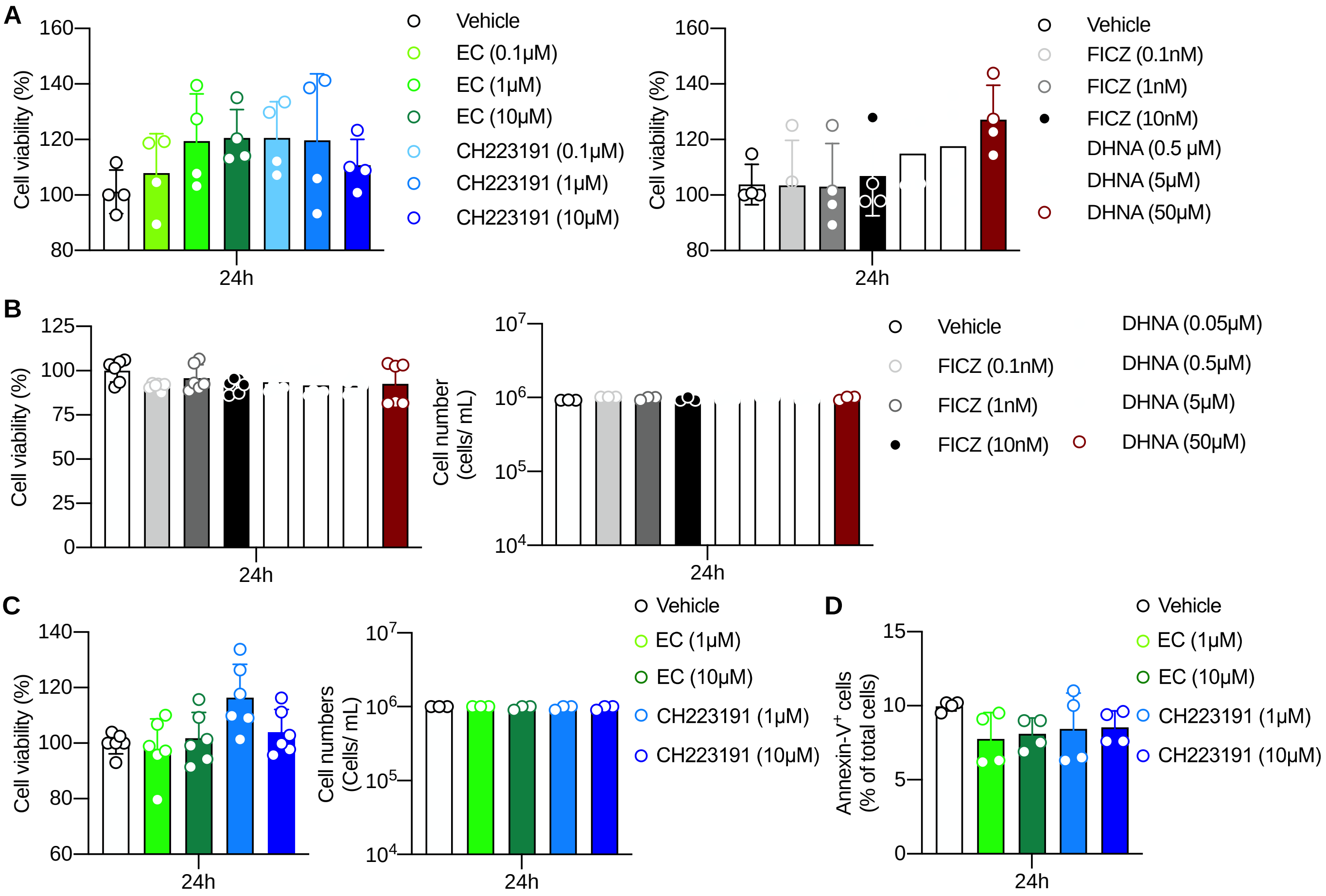

Supplementary Figure S1. Cell viability of ethyl caffeate treated HEXS34 cells or BMMCs. (**A**) After 24h treatment of EC, CH223191, FICZ, and DHNA, HEXS34 cell viability was evaluated by WST8 assay. (n=4). (**B**) After 24h treatment of FICZ and DHNA, BMMCs viability and cell numbers were evaluated by WST8 assay (n=6) and hemacytometer (n=3), respectively. (**C**) After 24h treatment of EC, CH223191, BMMCs viability and cell numbers were evaluated by WST8 assay (n=6) and hemacytometer (n=3), respectively. (**D**) After 24h treatment of EC, CH223191, Annexin-V$^+$ BMMCs were evaluated by FACS (n=4). Mean ± SD is shown. Statistical differences were determined by One-way ANOVA with Dunnett's post hoc test.

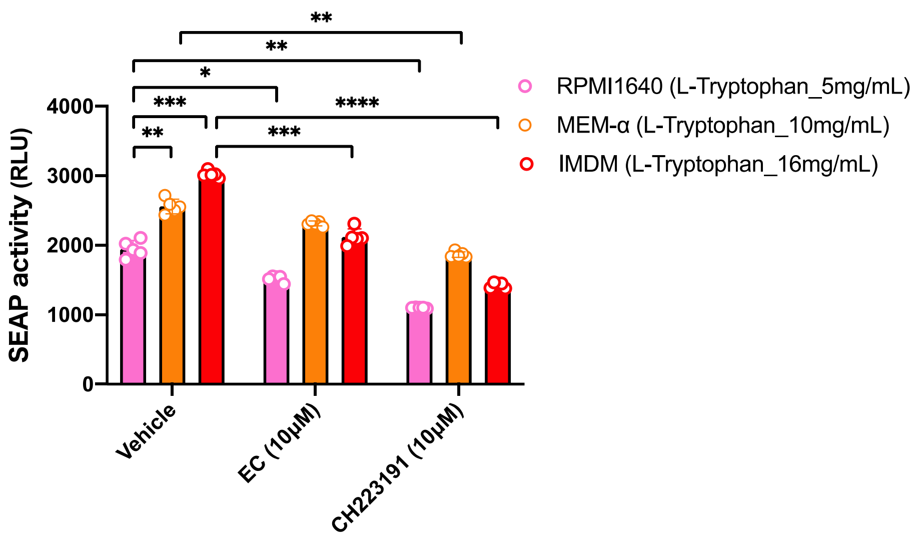

Supplement Figure S2. SEAP activity of IMDM-cultured HEXS34 cells was higher than MEM*α* or RPMI-cultured. After 24h replace culture medium to RPMI1640, MEM-*α* or IMDM, SEAP activity of HEXS34 cells were evaluated by chemiluminescence (n=5). Mean ± SD is shown. Statistical differences were determined by two-way ANOVA with Tukey's post hoc test, *P<0.05, **P<0.01, ***P<0.001, ****P<0.0001.

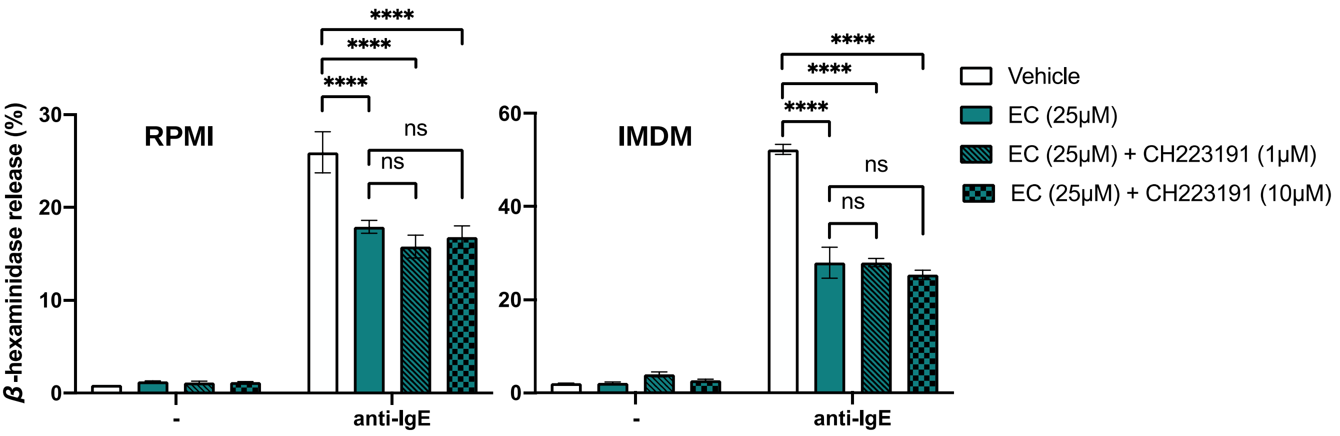

Suppl ement Figure S3. Ethyl caffeate and CH223191 share the same target. IgE-mediated release of β-hexosaminidase in EC treated-BMMCs with or without CH223191 for 12h (n=4). Mean ± SD is shown. Statistical differences were determined by two-way ANOVA with Tukey's post hoc test, ****P<0.0001, ns: not significant.
